# Idiopathic portal hypertension complicating systemic sclerosis: a case report

**DOI:** 10.1186/1471-230X-5-16

**Published:** 2005-05-26

**Authors:** John Moschos, Grigoris I Leontiadis, Clive Kelly, James Henry, Savvas Kadis

**Affiliations:** 1 Department of Gastroenterology, Queen Elizabeth Hospital, Sheriff Hill, Gateshead NE6 9SX, UK; 2 Department of Rheumatology, Queen Elizabeth Hospital, Sheriff Hill, Gateshead NE6 9SX, UK; 3 Department of Pathology, Queen Elizabeth Hospital, Sheriff Hill, Gateshead NE6 9SX, UK

## Abstract

**Background:**

Patients with systemic sclerosis may develop mild abnormalities of liver function tests. More serious hepatic involvement has been well documented but is rare. Idiopathic portal hypertension had been reported only in a few female patients with systemic sclerosis.

**Case presentation:**

An 82-year-old man with known systemic sclerosis presented with melaena. Urgent gastroscopy revealed oesophageal varices, which re-started bleeding during the procedure and were treated ensocopically, with Sengstaken tube and glypressin. Liver function tests and coagulation were normal. Non-invasive liver screen (including hepatitis viral serology and autoantibodies) was negative. Ultrasound scan of the abdomen revealed a small liver with coarse texture and no focal lesion. Hepato-portal flow was demonstrated in the portal vein. The spleen was enlarged. A moderate amount of free peritoneal fluid was present. A CT scan confirmed the absence of portal vein thrombosis. One month following discharge the patient had a liver biopsy. Histological examination showed essentially normal liver tissue; there was no evidence of any excess inflammation and no features to suggest cirrhosis or drug-induced liver disease. Taking into account the above evaluation we concluded that the patient had idiopathic portal hypertension.

**Conclusion:**

Both male and female patients with systemic sclerosis may – rarely – develop idiopathic portal hypertension.

## Background

Systemic sclerosis (SSc) is a multisystem disease of unknown cause. The incidence of the disease is 10/million population per year. The female/male ratio is 4:1. A few cases of idiopathic portal hypertension (IPH) associated with SSc have been reported, all in females.

## Case presentation

We report a case of an 82-year-old man admitted to the hospital with melaena and dizziness on standing.

He had a past medical history of SSc diagnosed 3 years before and pulmonary fibrosis. He was on prednisolone and azathioprine. There was no history of liver disease or alcohol abuse.

On examination his blood pressure of 95/65 mmHg, pulse 90/min. He had bilateral fine inspiratory crackles. The abdomen was soft and non-tender. Rectal examination revealed melaena.

White blood count was 12,700/mm^3^, haemoglobin 10.9 g/dl, platelets 186,000/mm^3 ^and MCV 99.4 fl. Blood urea was 12.5 mmol/l, creatinine 68 μmol/l, electrolytes were normal, glucose 6.3 mmol/l. Serum alanine aminotransferase was 12 u/l, bilirubin 12 μmol/l, γ-glutamyl transferase 54 u/l, alkaline phosphatase 35 u/l, total protein 52 g/l, albumin 27 g/l and C-reactive protein 12. Coagulation studies were normal. Factor V Leiden mutation was not present. Hepatitis viral serology and autoantibody screen were negative and α-1-antitrypsin was normal. Ferritin was 103 μmol/l, caeruloplasmin was 0.25 μmol/l and α-fetoprotein was 0.9 kU/L.

Urgent gastroscopy revealed oesophageal varices, fresh blood and clots in the stomach. During the procedure the oesophageal varices started bleeding. Haemostasis was not achieved despite sclerotherapy and therefore a Sengstaken tube was inserted. The patient was admitted to the High Dependency Unit and commenced on intravenous glypressin, antibiotics and was transfused four units of blood. The following day a repeat gastroscopy showed two columns of grade II oesophageal varices, two oesophageal ulcers (probably secondary to sclerotherapy) and mild portal gastropathy.

Ultrasound scan of the abdomen revealed a small liver with coarse texture and no focal lesion. Hepato-portal flow was demonstrated in the portal vein. The common bile duct was of normal caliber. The spleen was enlarged (bipolar length 12.8 cm). A moderate amount of free peritoneal fluid was noted. CT scan confirmed the absence of portal vein thrombosis.

The patient was discharged three weeks following admission on propranolol 40 mg twice daily, azathioprine 50 mg twice daily, spironolactone 100 mg once daily, prednisolone 7.5 mg once daily, lansoprazole, vitamin D and calcium. He had two further endoscopies, one and five weeks following discharge and his varices were successfully treated with banding. One month following discharge he had a liver biopsy. There was no evidence of any excess inflammation and no features to suggest cirrhosis or drug-induced liver disease. Overall this was essentially normal liver tissue.

Taking into account the liver histology and the absence of evidence of portal vein thrombosis on imaging we concluded that the patient had IPH. We decided not to proceed to portal pressure measurement because this would not contribute to the management of the patient.

Two months following discharge the patient was reviewed and was well. He had not had any further episodes of haematemesis or melaena. He had normal full blood count and biochemistry results.

## Conclusion

Abnormalities of liver function occur frequently in patients with rheumatic conditions. Although benign extra-articular manifestations of rheumatic disease are the most common ones, more serious hepatic involvement, including hepatic steatosis, vasculitis, nodular regenerative hyperplasia and primary biliary cirrhosis, portal vein obliteration and portal hypertension and rarely portal fibrosis with abnormal lobular architecture have been observed in rheumatic diseases. Vascular disorders of the liver also have been described, such as Budd-Chiari syndrome [1]. The medical management of rheumatic disease involves potentially hepatotoxic medications. Occult hepatitis C infection and associated cryoglobulinaemia can mimic rheumatic disease and antiviral therapy may be beneficial [2].

IPH is frequently associated with autoimmune diseases in Japan. In a large survey by questionnaire 12% of patients with IPH had one or two autoimmune diseases and a quarter of them had hyperglobulimaemia [3]. This data raise the possibility of causal rather than an incidental association between IPH and autoimmune disorders. An immunological basis for IPH pathogenesis has been suggested by Terada et al in Japan; the smaller venous radicles in the small and medium-sized portal tracts may be targets of immunologic attack in IPH [4].

Liver involvement in patients with SSc has been well documented but is rare. In a post-mortem series of 57 SSc patients histological liver damage was found in 8.8% of cases [5].

There are only a few reported cases of SSc with IPH, and all have been females [6-8]. Our case is the first male patient reported with SSc and IPH.

When causes of portal hypertension like cirrhosis, extra-hepatic portal vein obstruction, and infections like Schistosomiasis have been excluded, there remains a group of patients with portal hypertension of uncertain aetiology. These patients have good parenchymal reserve and much better survival than patients with portal hypertension caused by cirrhosis. Infection or exposure to several environmental toxins such as arsenic has been suspected as a cause but not proven.

There is a striking geographic variability in incidence and manifestations of IPH. In the United States, the sex incidence is nearly equal and the patients are generally above 50 years old, with gastrointestinal bleeding being the usual presentation. Liver function tests are normal and anaemia and leucopoenia are frequent. IPH is more common in India and Japan [9,10]. The largest study from India [9] observed female preponderance (62% female); mean age of presentation was 31 years. The majority of patients in this study presented with self-felt abdominal mass (splenomegaly) and / or gastrointestinal bleeding. In Japan 75% of patients are female, and the mean age of presentation is 36 years. In these patients gastrointestinal bleeding, splenomegaly, or anaemia are equally frequent.

The liver may look nodular, but this nodularity is usually relatively superficial. The nodules are similar to those seen in nodular regenerative hyperplasia. Nodularity is more pronounced in the cases from India. Cirrhosis by definition is not present, but there is usually significant fibrosis along the portal vein and its branches particularly near the hilus. However this is difficult to be recognized by liver biopsy. The portal vein usually has a thick sclerotic wall and there may be concentric fibrosis in the portal areas in the advanced cases. There may be diminution or obliteration of portal vein branches within the triads. Inflammation and necrosis are not prominent features of this condition. Portal vein thrombosis can occur, but this is a complication rather than a cause of IPH since it usually occurs later in the course of well-established disease.

In conclusion, clinicians should be aware of the rare association of systemic sclerosis and idiopathic portal hypertention, which may develop even in male patients.

## Competing interests

The author(s) declare that they have no competing interests.

## Authors' contributions

JM and SK drafted the manuscript., GL, CK and JH participated in the manuscript preparation. All authors were involved in the management of the patient and approved the final manuscript.

## Pre-publication history

The pre-publication history for this paper can be accessed here:


